# Tissue Expander-Based Breast Reconstruction at a Major Safety-Net Hospital: Managing the Outsized Risk of Infection

**DOI:** 10.1093/asjof/ojac036

**Published:** 2022-05-02

**Authors:** Lindsey N Urquia, Silas P Henderson, Jordyn T Farewell, Sofia Duque, Maycie Garibay, Julia Nevin, Andrew Y Zhang

**Affiliations:** Department of Plastic Surgery, University of Texas Southwestern Medical Center, Dallas, TX, USA; Department of Plastic Surgery, University of Texas Southwestern Medical Center, Dallas, TX, USA; Department of Plastic Surgery, University of Texas Southwestern Medical Center, Dallas, TX, USA; Department of Plastic Surgery, University of Texas Southwestern Medical Center, Dallas, TX, USA; Department of Plastic Surgery, University of Texas Southwestern Medical Center, Dallas, TX, USA; Department of Plastic Surgery, University of Texas Southwestern Medical Center, Dallas, TX, USA; Department of Plastic Surgery, University of Texas Southwestern Medical Center, Dallas, TX, USA

## Abstract

**Background:**

Immediate tissue expander (TE) breast reconstruction is reported to have the highest rate of postoperative infection among reconstructive modalities. The risk of infection is higher among patients treated at safety-net hospitals.

**Objectives:**

The goal of this study was to identify significant contributing factors to the elevated infection risk at our major safety-net institution.

**Methods:**

A retrospective chart review was conducted on all TE-based reconstruction patients with a diagnosis of postoperative infection between 2015 and 2019. Preoperative, perioperative, and postoperative risk factors for infection were determined and compared across patient and procedure demographics.

**Results:**

Two hundred forty-three patients, for a total of 412 breast reconstructions, were included in our study. Significant preoperative selection factors were identified to contribute to the elevated risk of infection, including the following: older age, higher BMI, and diabetes. Significant intraoperative and postoperative contributing factors included greater mastectomy weight, larger TE’s and intraoperative fill volume, and longer drain duration. Doxycycline treatment for infected patients resulted in a significantly higher rate of resolution.

**Conclusions:**

Safety-net hospital population patients undergoing TE breast reconstruction are at higher risk for postoperative infection. Personal and procedural risk factors are identified. Balancing the benefits of immediate breast reconstruction with TEs with the elevated risk of postoperative infection remains challenging. Implementation of more stringent eligibility criteria may help mitigate the risk of infection.

**Level of Evidence: 4:**

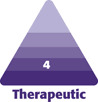

Postoperative infection remains a common and potentially devastating complication following prosthetic-based breast reconstruction after oncologic mastectomy.^1^ Of all breast reconstruction techniques currently available, immediate tissue expander (TE) reconstruction has been reported to have the highest rate of infection.^2^ The consequences of TE-associated infection can be significant, resulting in delays in cancer treatment, costly hospital admissions, poor aesthetic outcomes, and complete failure of the reconstructive process.^3,4^ Further, there is evidence to suggest that patients at safety-net hospitals are more prone to poorer surgical outcomes which may complicate breast reconstruction in this subset population.^5^ Despite these potential adverse outcomes, 2-stage reconstruction with immediate TE placement followed by definitive implant or autologous tissue reconstruction continues to be a mainstay of breast reconstruction due to its generally flexible timetable, positive aesthetic outcomes, and psychological benefit.^6,7^

In the preoperative time period, patients are presented with population-based percentages of risk for adverse outcomes of varying types of breast reconstruction options. A transparent discussion of risks, including the likelihood of postoperative infection, is crucial to the informed consent process. This conversation is made challenging by heterogeneity in the literature regarding postoperative complications within this patient population, with reported infection rates ranging from less than 1% to greater than 40%.^8,9^ Previous studies have scrutinized risk factors that predispose patients to develop periprosthetic infections such as increased age, higher BMI, diagnosis of diabetes, mastectomy specimen weight, and history of radiation therapy (XRT).^10^ With this information, numerous scoring systems have been developed in an attempt to more precisely calculate an individual’s risk for adverse outcome.^11,12^

Unfortunately, there remains no clear consensus in the literature regarding patient selection criteria, intraoperative infection control strategies, or treatment choice and duration for postoperative infection. For patients with more severe infections requiring hospitalization for intravenous (IV) antibiotics, empiric use of vancomycin with piperacillin-tazobactam (Zosyn [Pfizer, New York, NY]) is advocated,^2,3,13^ but despite their use, the risk for acute kidney injury (AKI), a major adverse effect from this combination,^14^ is not well documented in this population.

Our current preoperative risk stratification qualifies patients for immediate TE reconstruction at time of mastectomy based on abstaining from smoking for at least 4 weeks preoperatively, BMI less than 40 kg/m^2^, and diabetes control as indicated by a HbA1c level less than 8.0%. Our intraoperative infection control measures include use of chlorhexidine skin prep and a preoperative dose of IV cefazolin, re-dosed every 4 hours intraoperatively. Following mastectomy and prior to TE placement, the operative site is re-prepped with betadine, fresh drapes are placed around the surgical site, and the TE implants and acellular dermal matrix (ADM), Alloderm (LifeCell Corp., Bridgewater, NJ) are soaked in triple antibiotic solution (1 g cefazolin, 50,000 U bacitracin, 50 mg gentamicin in 1 L saline). The breast pocket is then irrigated with half strength betadine and triple antibiotic solution followed by placement of the TE and ADM using a no-touch technique. Two surgical drains are placed in each breast. Several of these interventions have been demonstrated to decrease the risk of postoperative TE infection.^15^ Following TE placement, patients are prescribed cephalexin for the duration of drain placement. Drains remain in place until their output is less than 30 mL per 24-hour period. Tissue expansion is begun approximately 3 weeks following TE placement, unless a TE infection is suspected. Suspected seroma is treated with ultrasound-guided aspiration and drain placement. For patients who develop TE infections, empiric outpatient antibiotic selection varies and is dependent on provider and patient antibiotic use history. Unless culture-specific information is available, vancomycin and Zosyn are empirically used in infections requiring IV antibiotics. In this current study, we examine our subset of breast reconstruction patients from a large safety-net hospital who underwent immediate TE-based reconstruction using the practices highlighted above and identify areas for improvement in the prevention and management of TE infections.

## METHODS

A retrospective chart review was conducted on all patients who underwent breast reconstruction following mastectomy at a large, single county safety-net hospital between January 2015 and December 2019. Because the data were retrospectively collected and de-identified, written patient consent for use of data was not required. Patients who underwent immediate TE-based reconstruction at time of mastectomy were included in this study. Data were collected and managed within a Research Electronic Data Capture (REDCap, Vanderbilt University, Nashville, TN) database hosted at our home institution.^16^ Patient demographics were collected and included medical and oncologic history, mastectomy procedure details, drain duration, and tissue expansion details. Surgical technique included subpectoral and prepectoral approaches, both with and without the use of ADM. Postoperative complications were recorded if the complication occurred any time before conversion to definitive implant or autologous reconstruction. TE reconstruction was deemed failed if the patient required explantation attributable to a postoperative complication.

Infection complication was ascribed to patients who met the following clinical criteria: abnormal erythema with one or more other clinical signs of infection (including but not limited to fever, chills, purulent drainage, warmth, or pain) that required the initiation of outpatient antibiotics beyond what was prescribed for the duration of drain placement, or hospital admission for IV antibiotic treatment and/or operative management for the aforementioned clinical signs of infection. For those who met one of these criteria, antibiotic selection, the need for hospitalization, and the incidence of hospital-acquired AKI were determined. AKI was defined using the Kidney Disease: Improving Global Outcomes (KDIGO) guidelines of an increase in creatinine by ≥0.3 mg/dL within 48 hours, an increase in creatinine to ≥1.5 times baseline, or a urine volume of <0.5 mL/kg/h for 6 hours.^17^

Statistical significance was compared using a *P*-value of <0.05 and a 95% confidence interval. A chi-squared test was utilized to compare proportions between 2 groups, and a *t* test was used to compare means between groups.

## RESULTS

Two hundred forty-three patients, with a total of 412 breast reconstructions performed from 2015 to 2019, were included in our study (Table 1). Our population had an average age of 46.6 ± 8.8 years and an average BMI of 30.4 ± 5.9 kg/m^2^. Nine percent of the included patients carried a diagnosis of diabetes and 3.3% were current smokers. Two hundred thirty-eight breasts (57.8%) had known oncologic lesions at the time of mastectomy while the remainder were performed prophylactically. Three hundred nineteen (77.4%) mastectomies were skin sparing, 87 (21.2%) were nipple sparing, and 6 (1.5%) were indeterminate; the mean mastectomy specimen weight was 740 ± 412 g. The majority of TEs were placed in a subpectoral position (86.4%) and utilized ADM (91.5%). The average intraoperative TE fill was 273 ± 159 mL. The average drain duration was 22.9 ± 10.5 days (Table 2).

**Table 1.** Summary of Patient Demographic Data, Medical History, and Oncologic History

Variable	All	Infection	No infection	*P*-value
Patients, n (%)	243 (100)	69 (28.4)	174 (71.6)	n/a
Age ± SD, years	46.6 ± 8.8	48.4 ± 8.5	45.9 ± 8.9	0.047
BMI ± SD, kg/m^2^	30.4 ± 5.9	31.9 ± 6.2	29.8 ± 5.8	0.013
Current smoker, n (%)	8 (3.3)	2 (2.9)	6 (3.5)	0.815
Diabetes, n (%)	21 (8.6)	10 (14.5)	11 (6.3)	0.041
Hypertension, n (%)	68 (28.0)	22 (31.9)	46 (26.4)	0.390
Preoperative chemotherapy, n (%)	101 (41.6)	24 (34.8)	77 (44.5)	0.168
Postoperative chemotherapy, n (%)	55 (22.6)	13 (18.8)	42 (24.3)	0.357
Preoperative radiation therapy, n (%)	5 (2.1)	2 (2.9)	3 (1.7)	0.552
Postoperative radiation therapy, n (%)	95 (39.1)	24 (34.8)	71 (40.8)	0.388

n/a, not applicable; SD, standard deviation.

**Table 2.** Summary of Oncological and Operative Details of Breasts That Underwent Mastectomy With Immediate Tissue Expander Placement

Variable	All	Infection	No infection	*P*-value
TEs, n (%)	412 (100)	76 (18.4)	336 (81.6)	n/a
Known oncologic lesion, n (%)	238 (57.8)	51 (67.1)	187 (55.7)	0.070
Skin sparing mastectomy, n (%)	319 (77.4)	63 (82.9)	256 (76.2)	0.208
Nipple sparing mastectomy, n (%)	87 (21.2)	13 (17.1)	74 (22.0)	0.345
Mastectomy specimen weight ± SD, g	740 ± 412	897 ± 434	704 ± 399	<0.001
Sentinel lymph node biopsy, n (%)	363 (88.1)	72 (94.7)	291 (86.6)	0.049
Axillary dissection, n (%)	59 (14.4)	13 (17.1)	46 (13.7)	0.445
Subpectoral TE placement, n (%)	356 (86.4)	68 (89.5)	288 (85.7)	0.383
TE capacity ± SD, mL	550 ± 126	607 ± 126	537 ± 122	<0.001
TE intraoperative fill ± SD, mL	273 ± 159	306 ± 163	265 ± 158	0.043
Acellular dermal matrix, n (%)	377 (91.5)	71 (93.4)	306 (91.1)	0.516
Surgical drain duration ± SD, days	22.9 ± 10.5	29.5 ± 15.2	21.5 ± 8.6	<0.001
Mean OR time (min)	269 ± 81	266 ± 77	270 ± 82	0.772

n/a, not applicable; OR, operating room; mL, milliliters; SD, standard deviation; TE, tissue expander.

### Patient-Specific Factors

**Figure 1.**
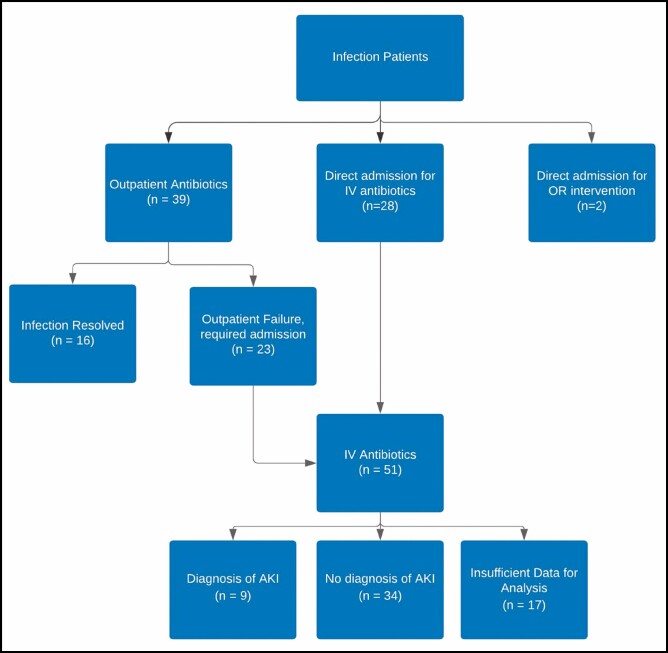
Tissue expander-associated infection outcomes. AKI, acute kidney infection; IV, intravenous; OR, operating room.

Sixty-nine patients, for a total of 18.4% of reconstructed breasts, received treatment for an infection based on the criteria described above (Figure 1). Compared to the patients without a diagnosed infection, patients within the infection group were significantly older (48.4 ± 8.5 vs 45.9 ± 8.9 years, *P* = 0.047), had a higher average BMI (31.9 ± 6.2 vs 29.8 ± 5.8 kg/m^2^, *P* = 0.013), and were more likely to carry an existing diagnosis of diabetes (14.5% vs 6.3%, *P* = 0.041). Preoperative smoking status was not statistically different between the 2 groups. There was no significant difference between preoperative or postoperative chemotherapy and XRT between the 2 cohorts. Table 3 demonstrates the infection rate per breast as it relates to patient BMI at varying cutoffs.

**Table 3.** Projected Influence of BMI Cutoffs on Relative Reduction in Infection Rate and Case Volume

BMI cutoff in kg/m^2^	TEs placed	TE infections	Infection rate	Relative risk reduction	Case volume reduction
≤28	152	19	12.5%	32.3%	63.1%
≤30	214	31	14.5%	21.5%	48.1%
≤32	268	39	14.6%	21.1%	35.0%
≤34	309	51	16.5%	10.6%	25.0%
≤36	338	56	16.6%	10.2%	18.0%
≤38	367	62	16.9%	8.5%	10.9%
Total	412	76	18.4%	n/a	n/a

n/a, not applicable; TE, tissue expander.

### Breast-Specific Factors

Compared to uninfected breast reconstructions, breasts that developed TE-associated infection were associated with mastectomy for radiographic or biopsy-proven cancer diagnosis (67.1% vs 52.4%), but this failed to reach significance (*P* = 0.070). They were, however, statistically more likely to occur on the same side as a concurrent sentinel lymph node biopsy (94.7% vs 86.6%, *P* = 0.049). Mean mastectomy specimen weight was greater in reconstructed breasts that developed infection compared to breasts that did not (897 ± 434 vs 704 ± 399 g, *P* < 0.001). Similarly, mean TE capacity was larger in the infection cohort (607 ± 126 vs 537 ± 122 mL. *P* < 0.001), as was mean TE intraoperative fill (306 ± 163 vs 265 ± 158 mL. *P* = 0.043). Drain duration differed significantly between the 2 groups; the mean drain duration within the infected cohort was 29.5 ± 15.2 days, and 21.5 ± 8.6 days in the noninfected group (*P* < 0.001). The use of ADM was not associated with increased risk for infection. Neither implant position in the prepectoral vs subpectoral plane nor mean intraoperative time was significantly different between these cohorts (Table 2).

### Postoperative Infection Treatment and Outcomes

Over the course of the study period, 69 patients were diagnosed with a TE infection. Of these patients, 23% were successfully treated with outpatient antibiotics alone. Overall, 74% of our TE infection patients required inpatient treatment with IV antibiotics, including those who failed outpatient treatment. Of those receiving inpatient management, 29% were successfully treated with IV antibiotics alone. The remaining patients required operative intervention for seroma complications, mastectomy flap necrosis, or infection complications; 6 implants were salvaged and 30 required explantation. Including the 2 patients who were directly admitted for explantation, we had an overall explant rate of 7.8% during the study period.

**Figure 2.**
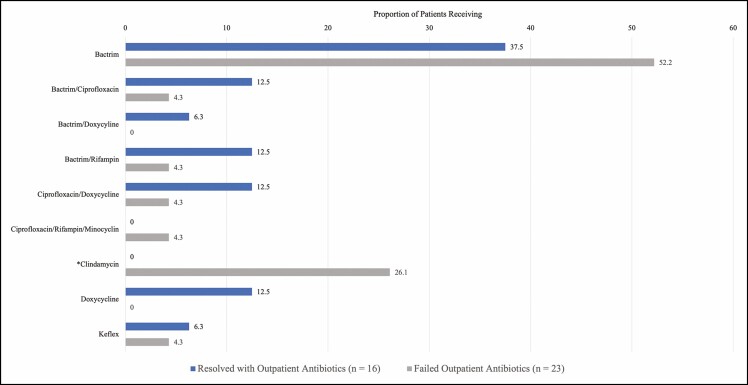
Differences in outcome in outpatient antibiotic regimens.

The most common outpatient regimen prescribed was monotherapy with trimethoprim-sulfamethoxazole, or Bactrim (Roche, Basel, Switzerland) (46.2%), followed by clindamycin monotherapy (15.4%). Of patients receiving outpatient antibiotics, 28.2% received combination therapy, most commonly Bactrim/rifampin, ciprofloxacin/doxycycline, and Bactrim/ciprofloxacin. The use of an antibiotic regimen containing doxycycline was associated with a higher rate of resolution with outpatient antibiotics alone (*P* = 0.024). Outpatient antibiotic failure rate was significantly higher in those receiving clindamycin monotherapy (*P* = 0.028). Patients who received Bactrim monotherapy had higher rates of hospital admission, although this difference was not statistically significant (*P* = 0.258) (Figure 2).

Patients admitted for IV antibiotics received regimens that were a combination of vancomycin, clindamycin, Zosyn, and/or ciprofloxacin. The majority (88.2%) received empiric vancomycin with Zosyn. Two-thirds (n = 34) of the IV antibiotic-treated patients had a creatinine level documented during the course of their treatment and were included in the statistical analysis. Of these, 9 (26.5%) met diagnostic criteria for AKI during hospital admission. The 2 groups had similar proportions of smokers, diabetics, and hypertensive patients and thus were considered comparable patient populations (Table 4). The cohort with documented AKI and the cohort without had no statistical difference in average age, BMI, or mean measured creatinine at the time of admission. At discharge, the mean creatinine in the AKI cohort was significantly greater than those without documented AKI complication (1.30 ± 0.31 vs 0.76 ± 0.14) (*P* < 0.001).

**Table 4.** Summary of Demographic Data, Medical History, and Oncologic History for Those Evaluated for Acute Kidney Injury During Admission for IV Antibiotics

Variable	All	AKI	No AKI	*P*-value
Patients, n (%)	34 (100)	9 (26.5)	25 (73.5)	n/a
Age ± SD, years	48.2 ± 9.5	45.5 ± 8.4	49.2 ± 9.8	0.322
BMI ± SD, kg/m^2^	32.1 ± 5.6	32.5 ± 6.3	31.9 ± 5.5	0.789
Smoking history, n (%)	3 (8.8)	2 (22.2)	1 (4.0)	0.104
Diabetes or prediabetes, n (%)	6 (17.6)	3 (33.3)	3 (12.0)	0.157
Hypertension, n (%)	13 (38.2)	3 (33.3)	10 (40.0)	0.727
Preoperative chemotherapy, n (%)	9 (26.5)	4 (44.4)	5 (20.0)	0.161
Postoperative chemotherapy, n (%)	5 (14.7)	2 (22.2)	3 (12.0)	0.465
Received outpatient antibiotics, n (%)	14 (41.2)	3 (33.3)	11 (44.0)	0.582
Vancomycin/piperacillin-tazobactam, n (%)	30 (88.2)	9 (100)	21 (84.0)	0.208
Length of admission ± SD, days	4.9 ± 2.2	6.7 ± 2.7	4.2 ± 1.6	0.002
Admission creatinine ± SD	0.72 ± 0.20	0.71 ± 0.27	0.73 ± 0.17	0.798
Discharge creatinine ± SD	0.90 ± 0.31	1.30 ± 0.31	0.76 ± 0.14	<0.001
IV antibiotic duration ± SD, days	4.0 ± 1.6	4.7 ± 1.7	3.8 ± 1.5	0.146

AKI, acute kidney injury; IV, intravenous; n/a, not applicable; SD, standard deviation.

All 9 patients within the AKI cohort and 84.0% of those in the non-AKI cohort were empirically managed with vancomycin and Zosyn during inpatient admission (*P* = 0.208). Patients who developed AKI had a longer average length of admission (6.7 ± 2.7 vs 4.2 ± 1.6 days, *P* = 0.002) compared to those without a documented AKI diagnosis. There was a correlation between duration of IV antibiotics and AKI (4.7 ± 1.7 vs 3.8 ± 1.5 days), but this failed to reach significance (*P* = 0.146).

**Figure 3.**
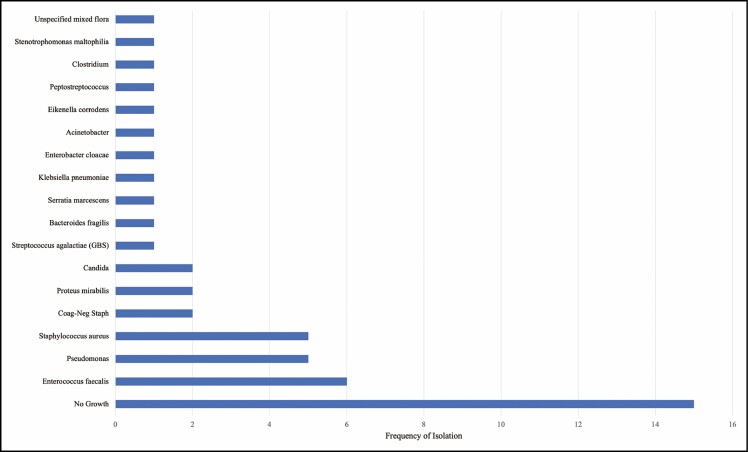
Microbiological species isolated from tissue expander-associated infections. GBS, Group B Streptococcus.

Periprosthetic fluid from operative washout, bedside aspiration, or percutaneous drain placement was collected and sent for bacterial culture in 70.6% of the admitted patients (n = 36). Cultures were positive in 58.3% of these patients (n = 21) and speciated a wide variety of organisms; 33.3% of the positive cultures yielded multiple organisms. The most frequently isolated organisms were *Enterococcus* (n = 6), *Staphylococcus aureus* (n = 5), and *Pseudomonas* (n = 5) (Figure 3). Notably, 3 *Staphylococcus* isolates were methicillin sensitive and 2 were methicillin resistant. Overall, gram-positive bacteria were isolated 16 times, gram-negative were isolated 14 times, and fungal pathogens were isolated twice. One culture isolated an unspecified mix of gram-positive and negative flora.

## Discussion

Postoperative infection remains a common complication following TE-based breast reconstruction and is a major impediment to successful reconstructive outcomes. Our study of 412 immediate TE breast reconstructions performed at a major safety-net hospital demonstrated an overall infection rate of 18.4% which is at the high end of published ranges; an average rate of 8.3% of breasts was recently reported in a meta-analysis (range 2.4%-20.0%).^1^

**Figure 4.**
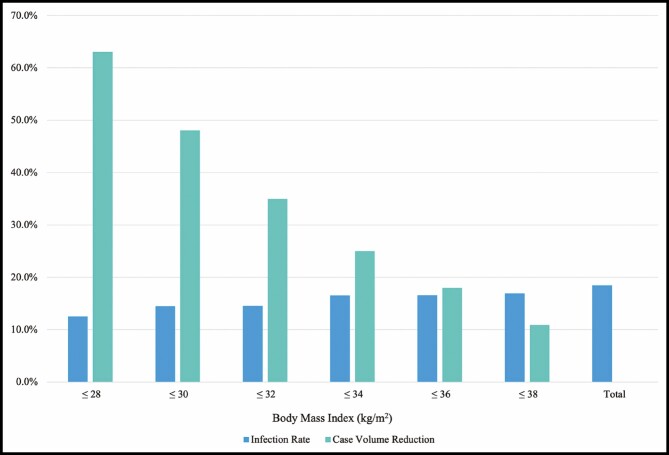
Relative reduction of tissue expander (TE) infection rate and TE case volume based on patient BMI.

One significant contributing factor to our higher-than-average infection rate is our preoperative selection criteria. The breast reconstruction population at our institution is known to have a higher BMI and higher percentage of diabetics compared to the national average,^1^ and several published studies, as well as our current study, demonstrate that patients with a higher BMI and with a diagnosis of diabetes are at a much higher risk of postoperative complications following breast surgery.^6,10^ It must be considered, on the other hand, that many studies clearly show significant improvements to a breast cancer patient’s well-being following breast reconstruction with TEs at the time of mastectomy.^6,7^ Therefore, balancing the risk of complications and the benefits of breast reconstruction at a large safety-net hospital with unfavorable population demographics becomes very challenging. While there is no clear recommended cutoff level for BMI and diabetes status in the literature, our criteria for breast reconstruction eligibility at the safety-net hospital are consciously loose to help combat the current disparities that persist with regards to access to breast reconstruction within this population.^18^ Internal research at our institution has already demonstrated success in this goal because a higher proportion of mastectomy patients opt to undergo breast reconstruction than at other safety-net hospitals,^19^ but this comes at the cost of including higher-risk patients. A stricter BMI cutoff is associated with a lower rate of infection complications as demonstrated within our study population (Table 3; Figure 4). If we, for example, disallowed immediate TE reconstruction in patients with a BMI greater than 32, we would expect to see a 21% relative risk reduction in infection complications at our institution at the cost of doing 35% less reconstructions overall.

Institutional constraints, such as limited operating room time and availability, and variability of mastectomy skin flap quality eliminate the feasibility of immediate autologous reconstruction at time of mastectomy. Therefore, for autologous breast reconstruction, we employ the delayed-immediate approach with TE placement at time of mastectomy followed by autologous reconstruction at a later date, which at our institution is always abdominally based. While the TE stage lessens the psychological impact of the patient’s mastectomy deformity and increases skin availability for the second stage of reconstruction, this delayed-immediate autologous approach has its downsides. The patients who generally qualify for abdominally based reconstruction tend to not only have higher BMIs but also larger breasts. Our study demonstrated that TE reconstruction in a breast with a larger mastectomy specimen weight, with a larger volume expander and greater intraoperative fill, had significantly higher rates of infection. This prompts the question of possibly pursuing delayed autologous reconstruction and avoiding TE reconstruction in these patients given their inherent risk factors.

Operative factors must also be taken into consideration when assessing a patient’s risk for infection. Our intraoperative infection management steps follow best-practice guidelines in existing literature and is currently the subject of ongoing quality improvement projects to discern the contribution of each element to the potential reduction of infection in our patient population. In our study, patients undergoing mastectomy in a breast with a known oncologic lesion appeared to be at a higher risk for infection. This may be directly correlated to mastectomy skin flap thickness and quality.^20^ The use of indocyanine angiography, which was not available during the study time period, may help mediate this issue.^21^ Neither the use of ADM nor TE pocket position was associated with a greater risk of infection in our study, a finding which is neither supportive of nor contradictory to current literature, because current studies regarding the 2 variables are heterogeneous in reported outcomes.^22-25^ Patel et al demonstrated increased overall complications with prepectoral TE placement, yet there was not an associated increase in infection complications, specifically, which has been supported by our study.^24^ Further research is necessary to describe the effect of both ADM and TE pocket position on infection rates within this patient population.

There are also opportunities for improvement in the perioperative and early postoperative periods. While our current drain practice detailed above is in line with the protocol published by Khansa et al in 2014,^15^ there is new evidence that this may no longer be best practice. Data from our study demonstrated a significantly longer duration of drains in the infection group compared to the cohort with no infection. Several studies, including Barbera et al in 2020, have demonstrated that drain removal at or prior to 21 days postoperatively regardless of drain output is a safe and effective strategy to mitigate infection complications in these patients.^26,27^

Additionally, newer literature is calling into question using prolonged prophylactic antibiotics in the postoperative period, with evidence demonstrating no added benefit but possible harm in the form of increased antibiotic resistance in those who do develop infection.^26,28-32^ Even though prophylactic antibiotics are still in use in our practice, based on our study findings, we have now switched from Keflex (Pragma Pharmaceuticals, LLC, Locust Valley, NY) to doxycycline postoperatively for broader coverage. Lastly, a notable proportion of our infections (37%) developed after the start of tissue expansion. Previous literature has suggested that expansion itself is not a major primary cause of TE infection,^33^ but given that more than one-third of our infections occur during the expansion stage, it would be beneficial for other studies at our institution to better ascertain this temporal relationship and evaluate the safety and efficacy of our current expansion practices.

There is currently no protocolized antibiotic treatment algorithm in place at our institution for patients who develop TE infection. Over the 5-year study period, there were 9 different outpatient regimens prescribed, ranging from Bactrim, doxycycline, or clindamycin monotherapy to various combination therapies. This study found that doxycycline use was associated with a higher rate of outpatient infection resolution, with only one patient who received a regimen containing doxycycline requiring admission. In contrast, all patients receiving clindamycin therapy failed outpatient treatment and subsequently required inpatient infection management. This differential is supported by our institution’s antibiogram which demonstrates superiority of doxycycline vs clindamycin in treating the gram-positive organisms most commonly isolated from these wounds. Current evidence indicates that gram-negative organisms are often insufficiently treated in the outpatient setting and are associated with an increased risk of TE loss.^13,34,35^ Broadening coverage with the addition of a fluoroquinolone has been suggested by several authors for the outpatient treatment of TE infections. This combination of antibiotics is tailored to the most commonly isolated organisms from these wounds, including coagulase-negative staphylococcus, *Staph aureus*, and *Pseudomonas*.^2-4^ Though not ascertained in this retrospective study, patient compliance should also be considered. Medication adherence is a complex phenomenon, yet through the lens of literature published on this subject, it is reasonable to infer that the simplicity of twice-daily doxycycline dosing vs clindamycin every 6 to 8 hours may lead to fewer missed doses and thus more efficacious drug concentrations during the treatment period.^36,37^

The rate of AKI diagnosis in our patient population exposed to IV antibiotics is of concern. Risk of AKI due to vancomycin and Zosyn exposure has been established repeatedly, with one meta-analysis reporting an AKI rate of 22.2% in patients receiving this regimen.^14^ Despite this risk, most physicians recommend the combination’s empiric use to cover methicillin-resistant *Staphylococcus aureus* (MRSA) and *Pseudomonas*, salvage reconstruction, and reduce morbidity.^2-4^ Culture results in our study population were notable for MRSA positivity in only 5.6% (n = 2) of all specimens collected. This statistic includes patients who received at least one dose of vancomycin prior to obtaining culture specimens and thus may underestimate the prevalence of MRSA positivity. Given the rate of AKI complications and low rate of MRSA-positive cultures at our institution, we now utilize empiric vancomycin in only those with significant risk factors for MRSA, positive MRSA surveillance screens, or patients with clinically worrisome examination findings. An alternative antibiotic combination for use in this patient population is cefepime with vancomycin which is associated with lower risk of AKI while maintaining broad gram-negative coverage.^38^

Thinking purely from an infection prevention perspective, a strong argument could be made to tighten our preoperative selection criteria for TE-based reconstruction, either denying reconstruction or offering delayed autologous reconstruction for patients with higher BMIs and poor diabetes control. This dilemma leaves several questions, though. What is an acceptable infection rate that is tolerated for the benefit of immediate reconstruction, and how stringent does the selection criteria need to be to achieve this rate? What would the impact of more stringent selection criteria be on patient satisfaction and the overall rate of breast reconstruction in our patient population? These are conversations that must be transparently discussed among providers and patients.

Our study is not without limitations. This study’s retrospective nature may be victim to inconsistencies in chart documentation and data collection. As a large safety-net institution, our patient population may disallow generalizations to the breast reconstruction population as a whole. The large range of TE infection rates found in the literature has been attributed to broad variations in how infections in breast reconstruction patients are defined.^9,39^ Our present study encompassed a broad definition of infection that, in addition to nonsterile periprosthetic fluid collections, included mild-to-moderate surgical site cellulitis because this has been documented to be a major risk factor for the development of implant-associated infection.^40^ Utilization of this broader inclusion criteria may in part explain the high rate of infection in our cohort. Accuracy in our assessment of AKI incidence was limited by the inconsistent frequency of documented creatinine values in the medical record, because 33.3% of admitted patients had only one creatinine measurement during hospitalization. Consequently, AKI incidence could potentially be higher than we have reported here. Potential confounding factors to this subgroup analysis include patient volume status, differences in IV fluid administration, infection severity, and individual variations in IV antibiotic dosing and frequency. A protocolized approach to order management in this patient population may aid in a more definitive clinical assessment.

## Conclusions

Patients undergoing TE-based breast reconstruction at safety-net hospitals are at higher risk for postoperative infection. Multiple patient-specific risk factors, including high BMI and poor diabetic control, and procedural risk factors, including drain duration, were identified. Balancing the benefits of immediate breast reconstruction with TEs and the elevated risk of postoperative infection in this population remains challenging.
